# Primary glia expressing the G93A-SOD1 mutation present a neuroinflammatory phenotype and provide a cellular system for studies of glial inflammation

**DOI:** 10.1186/1742-2094-3-2

**Published:** 2006-01-25

**Authors:** Kenneth Hensley, Haitham Abdel-Moaty, Jerrod Hunter, Molina Mhatre, Shenyun Mou, Kim Nguyen, Tamara Potapova, Quentin N Pye, Min Qi, Heather Rice, Charles Stewart, Katharine Stroukoff, Melinda West

**Affiliations:** 1 Free Radical Biology and Aging Research Program, Oklahoma Medical Research Foundation (OMRF), 825 NE 13^th ^Street, Oklahoma City, OK, 73104, USA; 2 Department of Cell Biology, University of Oklahoma Health Science Center (OUHSC), Oklahoma City, OK, 73104, USA; 3 University of Oklahoma College of Engineering, Bioengineering Program, Norman, OK, USA

## Abstract

Detailed study of glial inflammation has been hindered by lack of cell culture systems that spontaneously demonstrate the "neuroinflammatory phenotype". Mice expressing a glycine → alanine substitution in cytosolic Cu, Zn-superoxide dismutase (G93A-SOD1) associated with familial amyotrophic lateral sclerosis (ALS) demonstrate age-dependent neuroinflammation associated with broad-spectrum cytokine, eicosanoid and oxidant production. In order to more precisely study the cellular mechanisms underlying glial activation in the G93A-SOD1 mouse, primary astrocytes were cultured from 7 day mouse neonates. At this age, G93A-SOD1 mice demonstrated no *in vivo *hallmarks of neuroinflammation. Nonetheless astrocytes cultured from G93A-SOD1 (but not wild-type human SOD1-expressing) transgenic mouse pups demonstrated a significant elevation in either the basal or the tumor necrosis alpha (TNFα)-stimulated levels of proinflammatory eicosanoids prostaglandin E_2 _(PGE_2_) and leukotriene B_4 _(LTB_4_); inducible nitric oxide synthase (iNOS) and •NO (indexed by nitrite release into the culture medium); and protein carbonyl products. Specific cytokine- and TNFα death-receptor-associated components were similarly upregulated in cultured G93A-SOD1 cells as assessed by multiprobe ribonuclease protection assays (RPAs) for their mRNA transcripts. Thus, endogenous glial expression of G93A-SOD1 produces a metastable condition in which glia are more prone to enter an activated neuroinflammatory state associated with broad-spectrum increased production of paracrine-acting substances. These findings support a role for active glial involvement in ALS and may provide a useful cell culture tool for the study of glial inflammation.

## Introduction

Although the proximal cause of paralysis in amyotrophic lateral sclerosis (ALS) is the death of motor neurons, it is becoming widely accepted that motor neuron death in ALS is not cell autonomous but depends upon active and passive roles for ambient glial cells. The neuron-cell autonomy of ALS pathogenesis has been strongly questioned by a number of studies over the past several years. In work published during 2001, Rouleau et al. created a strain of transgenic mice that express mutant SOD1 specifically in neurons. These mice display no frank pathology even at 1.5 years of age [1]. Caroni's group subsequently reported similar findings [2]. Selective expression of mutant SOD1 only in astroglia, causes a type of astrogliosis but fails to produce motor neuron disease [3] in the absence of simultaneous mutant SOD1 expression in neurons. Nonetheless, Cleveland and colleagues recently showed that the rate of disease progression in mutant SOD1 chimeric mice depends on the extraneuronal expression of mutant SOD1 [4]. The survival of chimeric mice was dependent upon mutant SOD1 expression in neurons, but also highly dependent on the number of ambient mutant SOD1-expressing non-neuronal cells. These studies provide strong incentive to consider glial involvement in ALS.

With the advent of transgenic mouse models for familial amyotrophic lateral sclerosis (FALS), it has become more possible to study inflammatory and autoimmune features of the disease at distinct time points during the progression of the illness. Using the G93A-SOD1 mutant mouse model for ALS, Gurney et al. reported dramatically increased numbers of MHC-II^+ ^microglia and concomitant astroglial activation beginning prior to onset of paralysis and increasing during the paralytic phase [5]. Several recent studies have built upon these early studies by documenting reproducible, age-dependent elaboration of pro-inflammatory cytokines during the onset and progression phases of disease in the G93A-SOD1 mouse [6-11]. Tumor necrosis factor-α (TNFα) and its principle receptor TNF-RI are particularly elevated at pre- and post-symptomatic stages of disease [6-9], suggesting a rationale for the application of this cytokine in cell culture studies of ALS-linked glial activation. The time-course of cytokine up-regulation closely mirrors the time-course of protein oxidative damage, and begins approximately two weeks prior to the point of actual motor neuron death [7,12]. In addition to cytokines and reactive oxygen species, eicosanoids such as PGE_2 _are elevated and pharmacological antagonism of PGE_2_-synthesizing inducible cyclooxygenase (COX-II) improves prognosis in the murine model [13]. Likewise arachidonic acid 5-lipoxygenase (5LOX) is elevated in G93A-SOD1 spinal cords and the 5LOX antagonist nordihydroguaiaretic acid (NDGA) slows disease progression in the ALS mouse [14]. These findings suggest a robust, multi-faceted neuroinflammatory response, antagonism of which may slow the progression of ALS.

In order to better understand the contributions of astroglia to neuroinflammation in the ALS context, and to create a tool for the study of neuroinflammatory signal transduction, primary cortical astrocytes were cultured from neonatal mice bearing G93A-SOD1 mutations. The cells were characterized for their ability to synthesize salient biomolecules including cytokines, eicosanoids, and reactive oxygen species. G93A-SOD1 transgenic astroglia were found to synthesize higher-than-normal levels of TNFα, COX-II, 5LOX, and PGE_2 _even in the absence of deliberate stimulation. When challenged with TNFα alone or in combination with IFNγ, selective subsets of cytokines were further induced. Leukotriene B_4_, nitric oxide and protein oxidation increased more markedly in G93A-SOD1 glia challenged with TNFα or interferon-γ IFNγ than in similarly treated nontransgenic cells. Expression of high copy numbers of wild-type human SOD1 had no effect or slightly diminished the inflammatory indices. These findings suggest that SOD1 mutations fundamentally alter the phenotype of astrocytes, placing the cells in a metastable condition that is hypersensitive to certain types of ligand-induced activation.

## Materials and methods

### Animals

Mice expressing high copy numbers of human mutant G93A-SOD1 were obtained from Jackson Laboratories (Bar Harbor ME; strain designation B6SJL-Tg(SOD1 G93A)1Gur/J; [15-17]. In some control experiments mice were used that express equivalent protein levels of wild-type human SOD1 (B6SJL-TgN-(SOD1 G93A)-2Gur; Jackson Laboratories). Transgenic mice were maintained in the hemizygous state by mating G93A males with B6SJL-TGN females. All animal procedures were approved by the OMRF Institutional Animal Care and Use Committee (IACUC).

### Astrocyte culture

Primary mouse neocortical astrocytes were cultured by slight modifications of previously described methods [18] from G93A-SOD1 mice, matched nontransgenic littermates, or wildtype-human SOD1 expressing mice. In all cases the cortex was used to maximize astroglial yield. Briefly, the neocortex was removed from 7 day old pups under aseptic conditions and large blood vessels carefully removed. Tissue was rinsed and triturated in cold Ca^++^/Mg^+ ^free HBSS buffer, then centrifuged at 300 × *g *for five minutes. The resulting pellet was resuspended in 30 mL of 50% Dulbecco's Modified Essential Medium (DMEM) and 50% F12 media containing 10% heat-inactivated fetal bovine serum, 1% glutamine, and 1% streptomycin and penicillin. The 30 mL suspension was placed into a 75 cm^2 ^tissue culture flask. Cells obtained from individual mouse pups were plated in separate flasks. Media was replenished 7 days following the initial plating. Between 6–10 days after initial plating, glia became fully confluent at which time they were subcultured at a 1:4 dilution into 6- or 24-well plates. In all cases, unless otherwise specified, astroglial cultures were not futher subcultured. Furthermore each experiment compared genotype-specific cell responses between parallel cultures of identical passage number, obtained from paired neonatal pups (littermates in the case of G93A-SOD1 +/- and -/- mice). Paired cultures were prepared on the same day and subject to medium changes and manipulations in exactly parallel fashion so as to avoid artifacts arising from differences in medium self-conditioning, clonal selection, or other uncontrolled variables. Specific experiments were conducted deliberately on astroglia that had been subcultured intentionally for up to 5 passages. Statistically significant genotype-specific differences in cytokine-stimulated nitrite production and other variables, as indicated, were maintained at least to the fifth passage in these experiments. Purity of cultures was routinely assessed by immunocytochemistry using fluorescein-conjugated anti-OX-42 antibody (Chemicon, Temecula CA USA) to identify microglia, and rhodamine-conjugated rabbit anti-glial fibrillary protein (GFAP) antibody (Chemicon) to identify astroctyes.

### Cytokine treatments

In all experiments cell cultures were stimulated at full confluence (110,000 cells/cm^2^). Cells were treated with recombinant murine TNFα and/or interferon gamma (IFNγ) (BD Pharmingen, San Diego CA USA) as indicated in specific experiments. Cytokines were predissolved in 4% fatty acid-free bovine serum albumin (BSA) in 0.9% saline at 100-fold working concentration. Vehicle control treatments used 4% BSA: saline only. Because TNFα activity varied somewhat from lot to lot, each lot was pre-tested to determine the concentration of applied cytokine that would yield a measurable effect within the linear range of cell response. For cytokine treatments, culture medium was replaced with fresh medium. After 2 hours equilibration, cytokines or vehicle were diluted 1:100 into cell culture medium. Viability was routinely assessed by means of tetrazolium reduction assays (Aqueous OneStep^®^, Promega, Gaithersburg MD USA).

### Ribonuclease protection assays

Multiprobe ribonuclease protection assays (RPAs) were performed as described [14,7,8]. Cells or brain cortices were lysed in TRIzol™ mRNA isolation reagent (Life Technologies, Gaithersburg MD). Total RNA was quantified spectrophotometrically at 260 nm. Panels of mRNA were detected using commercial RPA kits (Riboquant™, Pharmingen, San Diego, CA). Radiolabeled probes were synthesized from DNA templates containing a T7 RNA polymerase promoter (Pharmingen). Templates were transcribed in the presence of 100 μCi [γ-^32^P]UTP to yield radioactive probes of defined size for each mRNA. Probes were hybridized with 5–10 μg total RNA, then treated with RNAse A and T1 to digest single-stranded RNA. Intact double-stranded RNA hybrids were resolved on 5% polyacrylamide/8 M urea gels. Dried gels were visualized using a phosphorimager (Molecular Dynamics, Sunnyvale CA) and bands quantified using instrument-resident densitometry software (ImageQuant™, Molecular Dynamics). Within each sample, the density of each apoptosis-associated mRNA band was normalized to the sum of the L32 + GAPDH bands.

### Eicosanoid assays

PGE_2 _and LTB_4 _were measured in cell culture medium using commercially available enzyme linked immunosorbent assays (ELISAs; Cayman Chemical, San Diego CA USA).

### Nitrite assay

Cell culture medium was assayed for NO_2_^- ^by the Griess assay as described [8]. Samples were mixed 1:1 with a mixture of equal portions sulfanilamide and napthylethylenediamine reagents (LabChem, Gaithersburg MD USA). External standards were prepared in fresh cell culture medium. The diazo product was measured spectrophotometrically at 560 nm.

### Western blots

Cells were lysed in 10 mM sodium acetate pH 6.5 containing 0.1% triton X-100, 100 μM sodium orthovanadate and 1:1000 diluted mammalian protease inhibitor cocktail (Sigma Chemical, St. Louis MO USA). After centrifugation, samples were assayed for total protein by Lowry assay [19], adjusted to constant concentration, mixed 1:1 with loading dye (50% glycerol, 10% Tris, 0.01% bromophenol blue) and electrophoresed across 4–20% gradient polyacrylamide gels. Protein concentration per well of confluent, matched cultures did not differ significantly amongst the genotypes (data not illustrated). Samples were electroblotted onto polyvinylidene difluoride (PVDF) membranes, blocked overnight in 4% BSA then probed with one of the following antibodies at 1:2000 dilution: rabbit polyclonal anti-iNOS (Chemicon); rabbit polyclonal anti-COX-II (Chemicon); mouse monoclonal anti-5LOX (Transduction Laboratories, Lexington KY USA); or mouse monoclonal anti-actin clone AC15 (Sigma Chemical) followed by the appropriate peroxidase-conjugated secondary antibody. Blots were developed using enhanced chemiluminescence (Amersham Biosciences, Buckinghamshire UK).

### Protein carbonylation

Protein carbonylation was measured using methods similar to those previously published [7]. Cells were lysed in 20 mM 2-(N-morpholino)-ethanesulfonate (MES) buffer pH 5.5 containing 0.1% triton X-100, 5 mM biotin-LC-hydrazide (Pierce Biotechnology, Rockford IL USA) and 100 μM butylated hydroxytoluene (BHT). Samples were incubated 2 H at 37°C, centrifuged, and supernatant was electrophoresed and blotted as describe above. Blots were blocked overnight in 4% BSA, probed with 50 ng/mL streptavidin-conjugated horseradish peroxidase (Pierce) and visualized by chemiluminescence. Control experiments omitted the biotin-LC-hydrazide reagent or substituted biotin for biotin-hydrazide; in neither case was any labeling observed.

### Statistics

Data were evaluated by analysis of variance (ANOVA) followed by *post-hoc *comparisons to assess genotype-specific differences in particular endpoints amongst nontransgenic, G93A-SOD1^+ ^and human wildtype-expressing glial cell cultures. All analyses were conducted using GraphPad Prism^® ^software (GraphPad, San Diege CA USA).

## Results

When fresh cortical tissue was excised from G93A-SOD1 and non-transgenic neonatal pups at 7 days of age, no genotype-dependent differences were observed with respect to PGE_2 _concentration as measured by ELISA (NonTg = 281 ± 200 pg/mg protein; G93A-SOD1 = 336 ± 134 pg/mg protein; N = 6/group); COX-II expression or 5LOX expression as measured by immunoblot (not shown); or cytokine expression patterns assessed by RPAs (data not shown). Nonetheless cultured astroglia demonstrated clear genotype-dependent differences in these several parameters, as described below.

Primary astrocyte cultures from G93A-SOD1 or nontransgenic mice were almost exclusively astrocytic based on immunocytochemical staining with anti-glial fibrillary acidic protein (GFAP) (not illustrated). In initial cultures, microglia were occasionally evident; however, these cells were not retained throughout multiple serial passages. Both nontransgenic and transgenic cells displayed typical morphological attributes of cultured astrocytes. G93A-SOD1 cells tended to be slightly more elongated than nontransgenic cells though no formal attempt was made to quantify or statistically analyze this feature. There was no discernible difference in rates of tetrazolium reduction amongst the genotypes, under any of the conditions tested. Viability of cells treated with maximum concentrations of stimulatory cytokines (40 ng/mL TNFα plus 50 U/mL IFNγ) did not differ significantly from that of untreated cells, based on tetrazolium reduction assays, at time points up to 48 hours post-stimulation.

### Specific cytokine expressioin differences occur in G93A-SOD1 astrocytes

**Figure 1:**
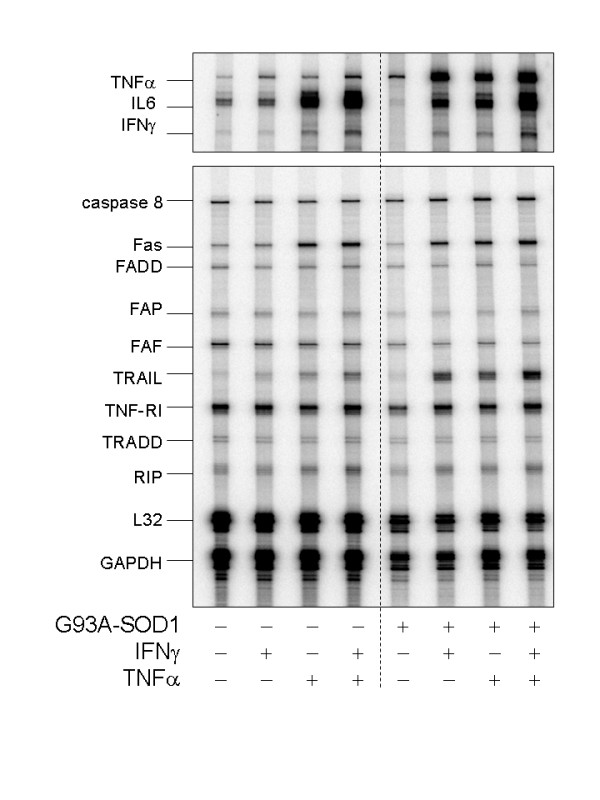
Representative multiprobe ribonuclease protection assay results indicating selective increase in certain cytokine mRNAs in G93A-SOD1 astrocyte cultures, either in the absence of deliberate stimulation (basal condition) or after 4 hours treatment with recombinant murine IFNγ (50 U/mL), TNFα (40 ng/mL) or both. Each lane represents pooled mRNA from at 6 wells of cells.

Multiprobe RPA methods were used to assess genotype-dependent differences in cytokine-stimulated cytokine expression between nontransgenic and G93A-SOD1 glia. Medium was replaced and cells were stimulated for 4 hours, which was found to represent the approximate peak for TNFα-stimulated cytokine mRNA transcription. A number of observations were evident in these experiments. First, G93A-SOD1 cells demonstrated lower levels of "housekeeping" messages L32 and GAPDH than did non-transgenic, matched cell cultures (Fig. 1). This may reflect a fundamental alteration in mRNA distribution with an over-expression of "non-housekeeping" genes such that the ratio of L32 and GAPDH to total mRNA, is fundamentally skewed in G93A-SOD1+ gial cultures. Thus, when equivalent amounts of message (based on UV absorption of RNA extracts) was loaded onto polyacrylamide gells, the cytokine: housekeeping message ratio could be noticeably affected.

With this possible qualification, G93A-SOD1 cells were found to (1) express more TNFα message in the basal state than did non-transgenic cells and (2) hyper-express TNFα message after either IFNγ or TNFα challenge (Fig. 1). TNFα stimulation produced, on average, 4-fold greater increase in TNFα message when G93A-SOD1 glia were stimulated than when nontransgenic cells were stimulated (% change in TNFα bands, without normalization to L32 + GAPDH = 1132 ± 618% in G93A-SOD1 cells vs. 242 ± 120% in nontransgenic cells, respectively, N = 5 experiments). TRAIL (TNF-Related Apoptosis-Inducing Ligand) was likewise very markedly upregulated in G93A-SOD1 cells following cytokine challenge, relative to nontransgenic cells (Fig. 1). Several other pro-inflammatory cytokines or apoptosis-related transcripts were differentially regulated in the G93A-SOD1 cells (Table 1). Numerous other transcripts did not differ notably in their levels as a function of genotype or stimulus (Fig. 1, Table 1). IL-6, which has some neuroprotective functions [20], tended to decrease in G93A-SOD1 cultures.

**Table 1:** Cytokine and apoptosis-associated message levels in nontransgenic and G93A-SOD1 astrocyte cultures in the basal (unstimulated) condition and 4 hours following stimulation with 50 U/mL IFNγ, 20 ng/mL TNFα or a combination of both cytokines. Data represent pooled samples from 6 wells in a typical experiment (see Fig. 1). Band intensities were normalized to the sum of GAPDH + L32 message levels prior to comparison between genotypes.

mRNA	Unstimulated G93A-SOD1 as % of unstimulated nonTg	Stimulated message level as % of unstimulated level, within genotypes					
		
		NonTg stimulated with:			G93A-SOD1 stimulated with:		
		
		IFNγ	TNFα	IFNγ + TNFα	IFNγ	TNFα	IFNγ + TNFα
Caspase 8	237	108	98	108	181	177	233
FADD	168	90	114	126	106	114	100
FAF	94	94	87	70	101	100	92
FAP	129	136	103	151	149	128	220
FAS	145	186	622	757	868	777	1392
IL1α	329	136	252	103	100	203	110
IL1β	644	152	1740	1101	161	724	223
IL1-RA	147	157	114	159	180	163	373
IL6	34	148	768	991	1461	1228	2755
IL18	307	221	104	92	199	205	194
IL12p35	341	141	239	263	108	71	103
IL12p40	325	121	137	165	197	94	1402
IFNγ	158	121	480	798	534	605	1792
MIF	70	83	129	133	95	110	108
RIP	159	130	152	225	230	161	258
TNFα	428	700	512	1511	2302	1669	3352
TNF-RI	166	132	99	131	180	112	176
TGFβ1	353	99	122	145	94	116	115
TGFβ3	155	90	96	87	94	132	148
TRADD	182	111	101	125	125	105	115
TRAIL	284	300	458	749	1307	734	1644

### Eicosanoid synthesis is increased in G93A-SOD1-expressing glia

**Figure 2:**
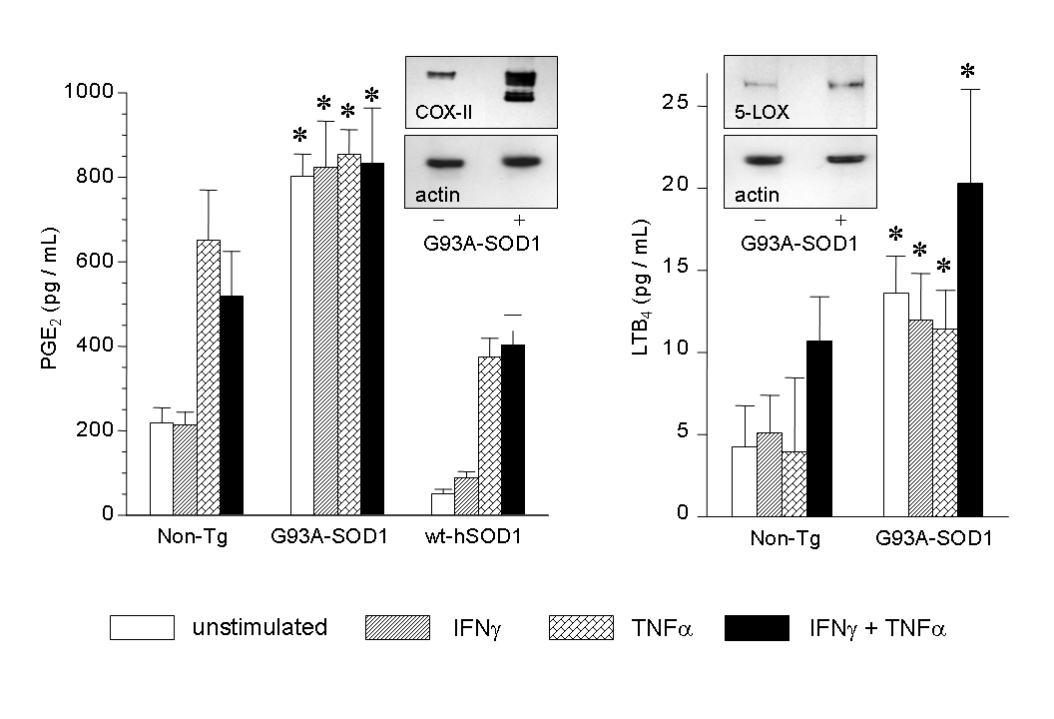
Comparison of basal and cytokine-stimulated PGE_2 _and LTB_4 _production by nontransgenic primary mouse astrocytes, G93A-SOD1 mouse astrocytes, or wild type human SOD1-expressing mouse astrocytes. Insets show western blot analysis of basal COX-II and 5-LOX protein expression. Data bars indicate mean ± SD of 6 wells of cells from a typical experiment. p < 0.05 overall by ANOVA; * indicates specific difference between nontransgenic and G93A-SOD1 cultures assessed by Bonferroni *post-hoc *tests.

Confluent, primary glia from G93A-SOD1 or nontransgenic neonatal mice, or from mice expressing high copy numbers of wildtype human SOD1 (wt-hSOD1) were stimulated with IFNγ, TNFα, or both for 24 hours and medium was assayed by ELISA for LTB_4 _and PGE_2_. As illustrated in Figure 2, PGE_2 _production was elevated three-fold in the G93A-SOD1 cells even in the absence of exogenous cytokines. The elevated production of PGE_2 _persisted through at least five serial passages of the astroglial cultures. COX-II protein was likewise increased in G93A-SOD1 glia. The G93A-SOD1 cells were resistant to additional cytokine-induced prostaglandin synthesis; however, the amount of PGE_2 _produced by unstimulated G93A-SOD1 glia was 2-fold greater than the amount that could be stimulated from nontransgenic cells by combined IFNγ plus TNFα (Fig. 2). A somewhat different pattern was observed for LTB_4_. Synthesis of this 5LOX metabolite was also elevated in G93A-SOD1 glia under basal conditions. However, LTB_4 _was synergistically inducible by IFNγ + TNFα in both genotypes (Fig. 2). The relative increase in LTB_4 _during cytokine stimulation was similar between the genotypes but LTB_4 _remained at least 2-fold elevated in G93A-SOD1 cultures relative to nontransgenic cultures, under all experimental conditions. These data begin to suggest fundamental perturbations to glial arachidonic acid metabolism as a function of the mutant SOD1 transgene.

### iNOS expression and nitric oxide synthesis is increased in G93A-SOD1 glia

**Figure 3:**
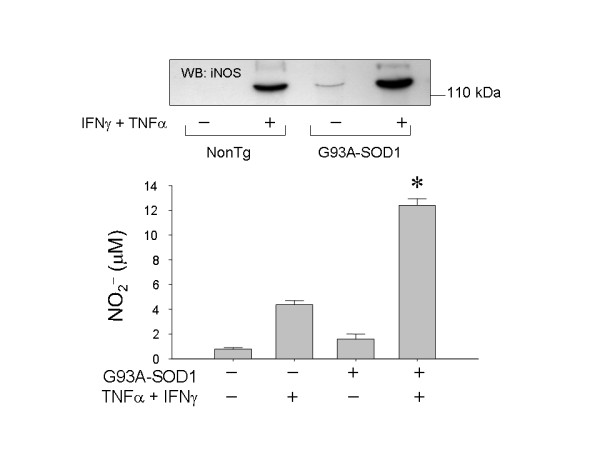
iNOS protein expression and NO_2_^- ^formation in cultured nontransgenic or G93A-SOD1+ astrocytes in the basal state and after 48 hours stimulation with recombinant murine TNFα (40 ng/mL) plus IFNγ (50 U/mL). Bars represent mean ± SD from 6 wells of cells in a typical experiment; * p < 0.05 for stimulated G93A-SOD1^+ ^cells relative to correspondingly treated nontransgenic cells, by two-tailed t-test.

Primary glia cultured from 7 day old G93A-SOD1 or nontransgenic pups were treated with increasing concentrations of TNFα plus or minus IFNγ. As an indicator of nitric oxide production, nitrite was measured in the cell culture medium 48 hours later. Measurable NO_2_^- ^formation required IFNγ in both non-transgenic and G93A-SOD1 astrocytes, and abundant nitrite production was only observed 48 hours after cytokine stimulation (Fig. 3). Under combined IFNγ and TNFα stimulation, TNFα-stimulated G93A-SOD1 glia produced significantly more NO_2_^- ^than did nontransgenic cells (Fig. 3). The G93A-SOD1 enhanced NO_2_^- ^production was maintained through at least 5 serial passages of cell cultures (not illustrated). Elevated levels of iNOS protein could be detected in G93A-SOD1 astrocytes relative to nontransgenic cells (Fig. 3).

### G93A-SOD1 astrocytes experience exacerbated protein carbonylation under cytokine challenge

Protein carbonyl accumulation is a well-accepted indicator of oxidative damage [7,20]. Recently biotin hydrazide and similar reagents have been adapted to monitor carbonylation in cell and tissue lysates [7]. The use of biotin hydrazide allows the sensitive detection of oxidized proteins by means of streptavidin conjugates, without resorting to antibody methods that are often hindered by low signal: noise and nonspecific binding artifacts. For these reasons, experiments were undertaken to assess genotype-related differences in glial protein carbonylation through means of the biotin labeling technique.

**Figure 4:**
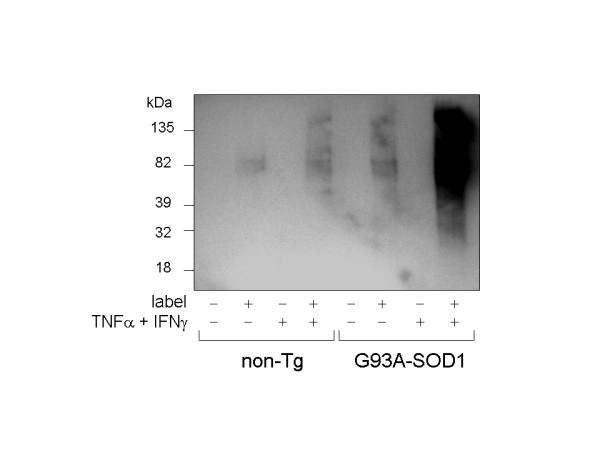
Basal and cytokine-stimulated protein carbonylation is increased in G93A-SOD1 astrocyte cultures. Cells were stimulated for 48 hours with 50 U/mL IFNγ plus 40 ng/mL TNFα, lysed in the presence or absence of biotin-LC-hydrazide (+ or - label as indicated), blotted onto a PVDF membrane and probed with streptavidin-conjugated horseradish peroxidase.

Cells were stimulated with 40 ng/mL TNFα plus 50 U/mL IFNγ, or vehicle for 48 hours and lysed for carbonyl assessment. The 48 hours timepoint was chosen as duration of treatment sufficient to induce obvious increases in protein carbonylation within nontransgenic astrocytes. The inclusion of IFNγ was also necessary to insure this effect. As shown in Fig. 4, G93A-SOD1 glia contained approximately 2-fold greater levels of protein carbonyl than did nontransgenic cells, in the absence of an applied cytokine challenge. After exposure to TNFα + IFNγ, protein carbonyl levels increased in both nontransgenic and G93A-SOD1 cells. Whereas the cytokine-stimulated increase in carbonylation was approximately 2-fold in nontransgenic cells, it was approximately 150-fold in G93A-SOD1 astroglia (Fig. 4; estimates for relative levels of carbonylation were made by repeated serial dilution of the labeled samples). Curiously, no major protein carbonylation band assignable to SOD1 was found in any G93A-SOD1 astrocyte lysates whereas a major carbonylated protein identifiable as SOD1 was previously demonstrated in spinal cord extracts from symptomatic G93A-SOD1 mice [7,12].

## Discussion

The role of astrocytes in paracrine inflammatory networks has become increasingly appreciated in recent years. In this capacity astrocytes likely respond to neural damage, infection, or tumorigenisis in such a way as to modulate necessary innate immune responses. Contrastingly, chronic unremitting neuroinflammation has been widely implicated in diverse neurodegenerative diseases. In murine models of ALS, neuroinflammation is robust as indicated by broad-spectrum cytokine upregulation plus oxidative stress, astroglial morphological changes, and microglial proliferation [6,14-12,5]. Aberrations in eicosanoid production, largely mediated by inducible cyclooxygenase-II (COX-II) [13] but perhaps also by 5LOX [14] represent another major component of the neuroinflammatory phenotype that might be amenable to therapeutic intervention. Thus far it has been difficult to separate the cell type-dependent contributions to the neuroinflammatory phenomenon. This limitation has prevented detailed molecular dissection of relevant pathways that are perturbed by the insertion of mutant SOD1 transgenes, and has slowed the development of new therapeutic modalities. The ability to recapture certain aspects of neuroinflammation in primary astrocyte cultures will likely facilitate detailed studies of signal transduction pathways that are sensitive to mutant SOD1.

The findings from the present study corroborate recent reports of cytokine hyper-expression in the CNS of mutant SOD1 mice preceding motor neuron death [6-8]. In particular the new data suggest that astrocytes cultured from 7 day old neonates reside in a metastable state that is exquisitely prone to activation, resulting in elevated expression of specific cytokines, upregulation of eicosanoid biosynthetic pathways, and increased oxidant production. The act of plating and culturing the cells seemed sufficient to induce expression of TNFα, COX-II and to a lesser extent 5LOX and iNOS. None of these inflammatory correlates were detectably elevated in cortical tissue extracted directly from the same transgenic neonates. Nonetheless, cultured glia from the same animals showed clear evidence for activation of the respective gene inductive pathways. Thus, glial over-expression of mutant SOD1 (but not wild-type SOD1) elicits a fundamental influence upon multiple gene regulatory pathways.

One of the most important, unaccomplished necessities in understanding ALS is to elucidate the toxic gain-of-function(s) inherent to SOD1 mutants. In this study we have demonstrated a cellular gain-of-function inasmuch as G93A-SOD1 fundamentally alters astrocyte response to relevant pro-inflammatory cytokines such as TNFα. Efforts are currently underway to discern the molecular mechanism(s) by which G93A-SOD1 alters glial sensitivity. One likely mode of action is through accumulation of mutant SOD1 within the mitochondrial intermembrane space [21,22] which may facilitate electron transport chain deficits, either directly or indirectly [23-26]. We have previously documented that mitochondrial inhibitors such as antimycin-A that disrupt electron transport, are sufficient to stimulate cytokine transcription in primary astrocyte cultures [27]. Thus factors including, but not restricted to reactive oxygen species may be released from glial mitochondria secondary to accumulation of mutant SOD1. These mitochondria-derived oxidants, lipids and proteins then can act through redox-sensitive mitogen-activated protein kinases [27] or directly upon transcription factors [28] to facilitate gene expression events thereby plausibly accounting for some of the hypersensitivity inherent to the G93A-SOD1 glial cultures. These concepts deserve closer scrutiny in future research and are under active investigation within our laboratory.

A major question that remains to be answered is whether or not increased cytokine and eicosanoid production in G93A-SOD1 central nervous system tissue, actually endangers ambient neurons. Most cytokines, including TNFα and IL6, that we find upregulated in primary glial cultures or *in vivo *[7,8], exert pleiotropic effects and can be trophic to pure neurocultures. In the presence of microglia however these cytokines trigger production of diffusible oxidants and could dysregulate key metabolic pathways, such as the kynurenine pathway, leading to production of excitotoxins (eg. quinolinic acid) and other paracrine factors that might injure neaby neurons [29]. Research ongoing in our laboratory is underway in attempts to address this issue.

## Competing interests

The authors declare they have no competing interests that impinge upon data presented within this manuscript.

## Authors' contributions

KH supervised the work presented in this manuscript. MW managed the mouse breeding program. QP and SM prepared and maintained cell cultures. H A-M, JH, MM, KN, TP, MQ, HR, and KS performed or assisted with biochemical assays for nitrite, eicosanoids, and immunoblots. CS performed ribonuclease protection assays.
